# Face cooling with mist water increases cerebral blood flow during exercise: effect of changes in facial skin blood flow

**DOI:** 10.3389/fphys.2012.00308

**Published:** 2012-08-02

**Authors:** Taiki Miyazawa, Masahiro Horiuchi, Daisuke Ichikawa, Andrew W. Subudhi, Jun Sugawara, Shigehiko Ogoh

**Affiliations:** ^1^Center for Biomedical Engineering Research, Toyo UniversityKawagoe, Japan; ^2^Research Institute of Industrial Technology, Toyo UniversityKawagoe, Japan; ^3^Department of Biomedical Engineering, Toyo UniversityKawagoe, Japan; ^4^Department of Biology, University of Colorado, Colorado SpringsCO, USA; ^5^Human Technology Research Institute, National Institute of Advanced Industrial Science and TechnologyTsukuba, Japan

**Keywords:** middle cerebral artery blood velocity, external carotid artery, internal carotid artery, laser Doppler flowmetry, diving reflex

## Abstract

Facial cooling (FC) increases cerebral blood flow (CBF) at rest and during exercise; however, the mechanism of this response remains unclear. The purpose of the present study was to test our hypothesis that FC causes facial vasoconstriction that diverts skin blood flow (SkBF_face_) toward the middle cerebral artery (MCA *V*_mean_) at rest and to a greater extent during exercise. Nine healthy young subjects (20 ± 2 years) underwent 3 min of FC by fanning and spraying the face with a mist of cold water (~4°C) at rest and during steady-state exercise [heart rate (HR) of 120 bpm]. We focused on the difference between the averaged data acquired from 1 min immediately before FC and last 1 min of FC. SkBF_face_, MCA *V*_mean_, and mean arterial blood pressure (MAP) were higher during exercise than at rest. As hypothesized, FC decreased SkBF_face_ at rest (−32 ± 4%) and to a greater extent during exercise (−64 ± 10%, *P* = 0.012). Although MCA *V*_mean_ was increased by FC (Rest, +1.4 ± 0.5 cm/s; Exercise, +1.4 ± 0.6 cm/s), the amount of the FC-evoked changes in MCA *V*_mean_ at rest and during exercise differed among subjects. In addition, changes in MCA *V*_mean_ with FC did not correlate with concomitant changes in SkBF_face_ (*r* = 0.095, *P* = 0.709). MAP was also increased by FC (Rest, +6.2 ± 1.4 mmHg; Exercise, +4.2 ± 1.2 mmHg). These findings suggest that the FC-induced increase in CBF during exercise could not be explained only by change in SkBF_face_.

## Introduction

Selective facial cooling (FC) increases middle cerebral artery mean blood velocity (MCA *V*_mean_) at rest (Brown et al., 2003) and during exercise (Kjeld et al., 2009). Interestingly, FC is particularly effective in improving endurance exercise performance in hot environments, e.g., increasing exercise time to fatigue (Ansley et al., 2008) and lowering ratings of perceived exertion (RPE) (Mundel et al., 2007; Ansley et al., 2008). This exercise performance improvement may be related to a FC-induced increase in cerebral blood flow (CBF) which may support brain neuronal activity and metabolism and reduce central fatigue during exercise (Ide and Secher, 2000; Secher et al., 2008; Ogoh and Ainslie, 2009). Although FC elicits the diving reflex in which facial cold receptors stimulate cardiovascular changes, including bradycardia and peripheral vasoconstriction (Fagius and Sundlof, 1986; Foster and Sheel, 2005; Kinoshita et al., 2006) that may divert blood toward the brain, the underlying mechanism of a FC-induced increase in CBF remains unclear.

Anatomically, the internal carotid arteries (ICA) and external carotid arteries (ECA) branch from the common carotid artery and supply blood to brain and facial regions, respectively. Through graded dynamic exercise, ECA blood flow and facial skin blood flow (SkBF_face_) gradually increases in an intensity-dependent manner (Sato et al., 2011). In addition, this increase in ECA blood flow is negatively correlated with the concomitant change in ICA blood flow (Sato et al., 2011), suggesting that an exercise-induced increase in ECA blood flow may restrict blood flow into the brain during exercise. Therefore, it is plausible that changes in ECA blood flow, i.e., SkBF_face_, contribute to CBF regulation independently of the diving reflex.

Given this background, the aim of this study was to examine whether carotid artery blood flow distribution (e.g., toward the brain and face) contributes to a FC-induced increase in CBF during exercise. We hypothesized that FC elicits a greater attenuation of SkBF_face_ during exercise, since a greater proportion of CBF may be directed to the ECA for thermoregulation, and thus we would measure a larger increase in MCA *V*_mean_ than at rest.

## Materials and methods

### Subjects and ethical approval

Nine healthy men with a mean (±SD) age of 20 ± 2 years, height of 170 ± 5 cm, and body mass of 62 ± 9 kg voluntarily participated in this study. Each subject provided written informed consent after all potential risks and procedures were explained. All experimental procedures and protocols conformed to the Declaration of Helsinki and were approved by the Institutional Review Boards of Faculty of Science Engineering, Toyo University (IRB # 2010-R-07). None of the subjects were taking any medication that may have influenced the hemodynamic responses to exercise. All subjects were familiarized with the equipment and procedures before any experimental sessions.

### Experimental design

On the experimental day, all subjects arrived at the laboratory in the morning 2 h after a light breakfast. The subjects were requested to avoid caffeinated beverages, alcohol, and strenuous physical activity for at least 24 h before the experiment. After the subjects were instrumented, they were seated in a semi-recumbent position with a backrest and rested quietly to allow for cardiovascular stability. In the Rest trial, after 3 min data collection (i.e., baseline at rest) the face of subject was cooled for 3 min by spraying the face with a mist of cold water (~4°C) and fanning with fan placed 50 cm from the subject's face and positioned facing upwards so as to cool only the head. In the Exercise trial conducted after securing sufficient time for the recovery from physiological changes induced by the Rest trial, subjects performed 4 min of an incremental warm up from the initial work load of 60 W at a pedal rate of 60 revolutions/min. The subjects were told to maintain the frequency of pedaling, and work load was increased 10–30 W every minute. With the target heart rate (HR: corresponding to 120 bpm) was achieved, workload was held constant (129 ± 12 W) for 5 min. After verification of steady-state—no increase in HR during the last 1 min of constant load cycling—each subject underwent 3 min of FC while continuing exercise. Subjects were instructed to breathe as normally as possible throughout the test. All the tests were carried out in a warm laboratory environment (27–28°C).

### Cardiorespiratory and RPE measurement

HR was monitored using a lead II electrocardiogram (ECG). Beat-to-beat mean arterial blood pressure (MAP) was measured using finger photoplethysmography (Finometer, Finapres Medical Systems BV, Netherlands). Stroke volume (SV) and cardiac output (Q) were estimated using the Modelflow method (Beat Scope 1.1, Finapres Medical Systems BV). This method provides a reliable estimate of changes in SV and Q in healthy humans from rest to submaximal exercise (Sugawara et al., 2003). Total peripheral resistance (TPR) was calculated as MAP divided by Q. Expired air was sampled breath-by-breath and end-tidal partial pressure of carbon dioxide (CO_2_) (P_ET_CO_2_) was measured with a gas analyzer system (AE-310S, Minato medical science co., Osaka, Japan). In the Exercise trial, RPE were recorded using the 15-point Borg scale (Borg, 1982) immediately before FC and the end of FC.

### Cerebral blood flow measurement

Mean blood flow velocity in the left middle cerebral artery (MCA *V*_mean_) was obtained by transcranial Doppler ultrasonography (Multidop T, DWL, Sipplingen, Germany). A 2-MHz Doppler probe was placed over the left temporal window and fixed with an adjustable headband. Cerebrovascular resistance index (CVRi) was calculated as MAP divided by MCA *V*_mean_. Relative change (%) in skin blood flow in the left forehead, approximately 3 cm from the midline and just above the supra-orbital ridge (SkBF_face_), was measured by using laser Doppler flowmetry (ALF21, Advance, Japan).

### Temperature measurement

Skin temperature was measured using thermistors (LT-ST08-12, Gram Co., Japan) placed on the center of the forehead (T_face_), the right side of the upper arm (T_arm_), chest (T_chest_), thigh (T_thigh_), and leg (T_leg_). Mean skin temperature (T_sk_) was calculated from the body surface area distribution and thermal sensitivity of each skin area using the following formula, which was proposed by Ramanathan (1964):

$${\text{T}}_{\text{sk}}=0.3\times ({\text{T}}_{\text{arm}}+{\text{T}}_{\text{chest}})+0.2\times ({\text{T}}_{\text{thigh}}+{\text{T}}_{\text{leg}})$$

### Data analysis

All measurement data were sampled continuously at 1 kHz using analog-to-digital converter (PowerLab, AD Instruments, Milford, MA) interfaced with a computer. Baseline data were obtained by averaging across 1 min immediately before FC at rest (i.e., baseline at rest) and from 4^th^ to 5^th^ min of exercise at constant workload (i.e., baseline during exercise). Averaged data were also acquired from 2^nd^ to 3^rd^ min of facial cooling at rest (i.e., FC at rest) and during exercise (i.e., FC during exercise).

### Statistics

Statistical analysis was performed using SigmaStat 3.5 software (Systat Software Inc., CA, USA). Following confirmation of distribution normality using Shapiro Wilk *W* tests, data were analyzed using a Two-Way (*Condition*: Baseline and FC) × (*State*: Rest and Exercise) repeated measures analysis of variance (ANOVA) with *post-hoc* Tukey's test. Student's paired *t*-tests were used to analyze influence of rest and exercise for the FC-induced changes of physiological parameters. Correlation coefficients were obtained to determine the relation between FC-induced changes in SkBF_face_ and MCA *V*_mean_. Data are expressed as mean ± S.E.M. with significance for all two-tailed tests set at *P* < 0.05.

## Results

Overall T_sk_ was significantly increased during exercise, but FC did not change T_sk_ at rest or during exercise. In contrast, T_face_ was decreased (Rest, −6.2 ± 0.3°C; Exercise, −6.9 ± 0.3°C), indicating that body parts other than face were not affected by FC (Table 1).

**Table 1:** **Skin temperature responses**.

	**Rest**		**Exercise**	
	**Baseline**	**FC**	**Baseline**	**FC**
T_face_	34.6 ± 0.2	28.5 ± 0.4^*^	35.6 ± 0.2^†^	28.7 ± 0.3^*^
T_sk_	33.9 ± 0.2	33.8 ± 0.2	34.8 ± 0.2^†^	34.7 ± 0.2^†^

Values are means ± S.E.M.; T_face_, forehead skin temperature; T_sk_, mean skin temperature.

* P < 0.05 different from baseline.

† P < 0.05 different from Rest.

During exercise MCA *V*_mean_, SkBF_face_, MAP, HR, SV, and Q were increased, whereas TPR was decreased and CVRi was unchanged (Table 2). In response to FC, SkBF_face_ decreased and MCA *V*_mean_, MAP, and SV increased at rest and during exercise. HR was decreased and TPR and CVRi were increased with FC at rest, but unchanged with FC during exercise. Q was unchanged with FC at rest and during exercise, but FC induced-change in Q tended to be higher during exercise (*P* = 0.08). P_ET_CO_2_ was significantly increased during exercise but was unchanged with FC at the both conditions. RPE was not affected by FC during exercise.

**Table 2:** **Hemodynamic and ventilatory responses and RPE**.

**Parameter**	**Rest**		**Exercise**	
	**Baseline**	**FC**	**Baseline**	**FC**
MCA *V*_mean_, cm/s	53 ± 3	55 ± 3^*^	58 ± 4^†^	60 ± 4^*^^†^
SkBF_face_, %	0 ± 0	−32 ± 4^*^	132 ± 43^†^	68 ± 36^*^^†^
MAP, mmHg	87 ± 3	93 ± 3^*^	101 ± 2^†^	105 ± 3^*^^†^
HR, bpm	64 ± 2	57 ± 2^*^	120 ± 3^†^	118 ± 3^†^
SV, %	0 ± 0	11 ± 3^*^	36 ± 6^†^	40 ± 7^*^^†^
Q, %	0 ± 0	−1 ± 2	157 ± 16^†^	161 ± 16^†^
TPR, mmHg/(L/min)	17.5 ± 1.1	18.8 ± 1.1^*^	7.9 ± 0.4^†^	8.0 ± 0.4^†^
CVRi, mmHg/(cm/s)	1.69 ± 0.10	1.77 ± 0.13^*^	1.78 ± 0.09	1.81 ± 0.10
P_ET_CO_2_, mmHg	44 ± 1	44 ± 1	49 ± 1^†^	49 ± 1^†^
RPE	−	−	11 ± 1	12 ± 1

Values are means ± S.E.M.; MCA V_mean_, middle cerebral artery mean blood velocity; SkBF_face_, forehead skin blood flow; MAP, mean arterial pressure; HR, heart rate; SV, stroke volume; Q, cardiac output; TPR, total peripheral resistance; CVRi, cerebrovascular resistance index; P_ET_CO_2_, partial pressure of end tidal carbon dioxide; RPE, rating of perceived exertion.

* P < 0.05 different from baseline.

† P < 0.05 different from rest.

**Figure 1:**
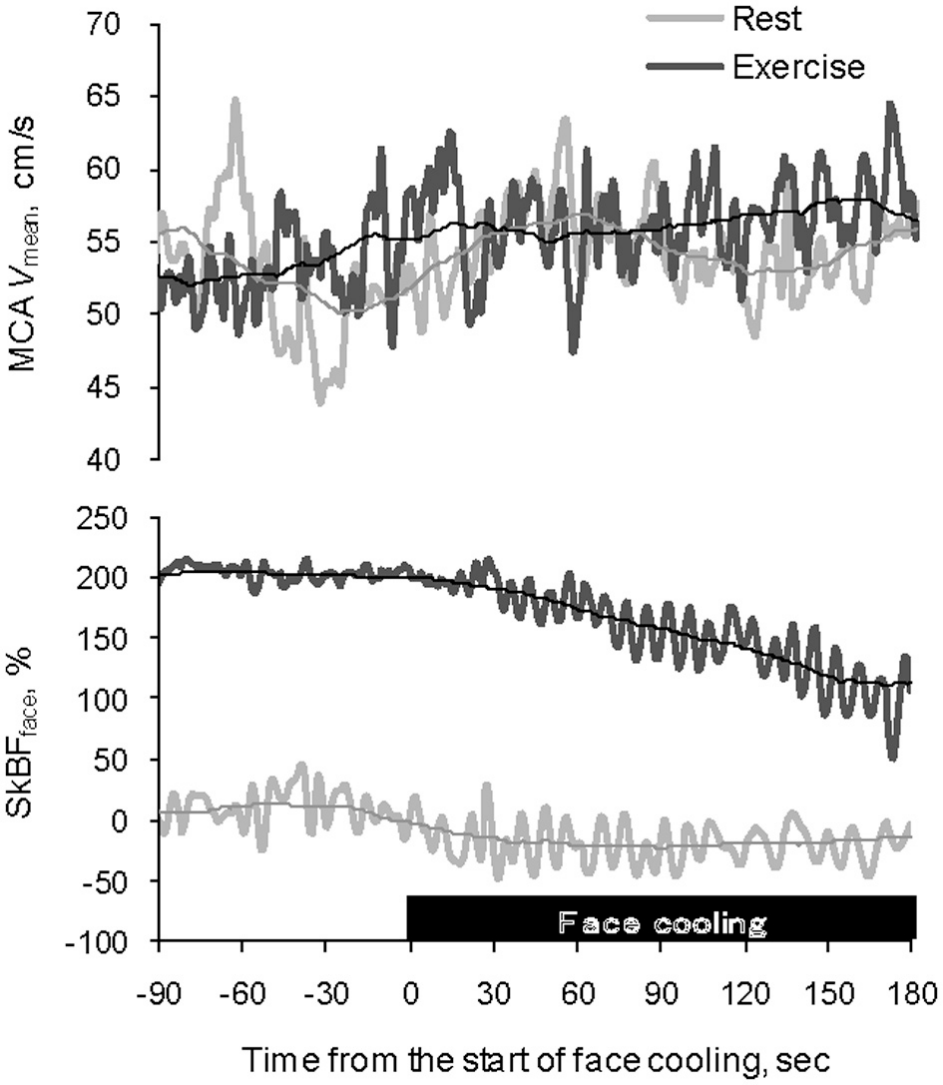
**Typical example of recordings of MCA *V*_mean_ and SkBF_face_ at rest (gray) and during exercise (black) showing the effect of facial cooling**. Each thin line represents a moving average for 1 min. MCA *V*_mean_, middle cerebral artery mean blood velocity; SkBF_face_, forehead skin blood flow.

**Figure 2:**
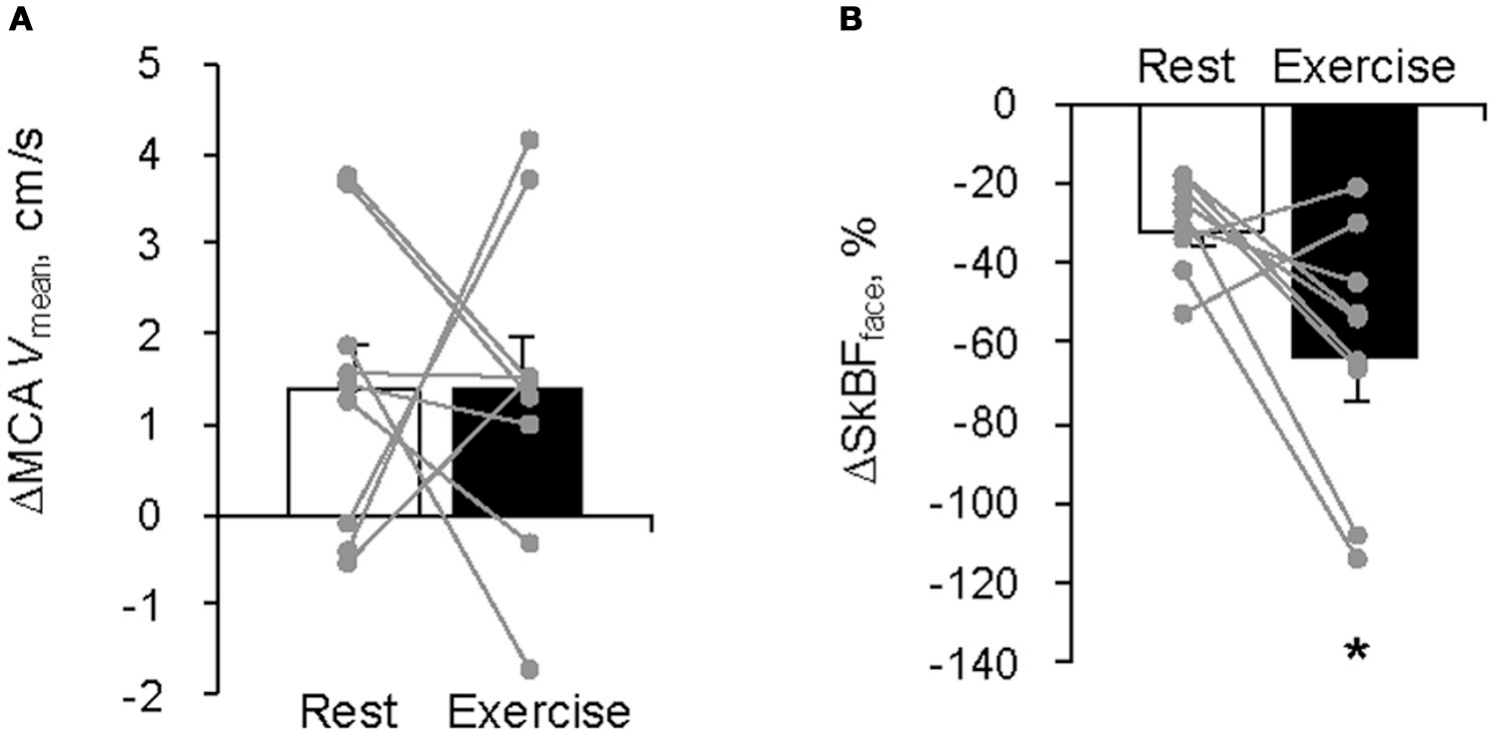
**Facial cooling-induced hemodynamic changes (Δ) at rest and during exercise: (A) middle cerebral artery mean blood velocity (MCA *V*_mean_) and (B) forehead skin blood flow (SkBF_face_)**. Each gray plot represents an individual response. Values are means ± S.E.M. ^*^P < 0.05 different from rest.

**Figure 3:**
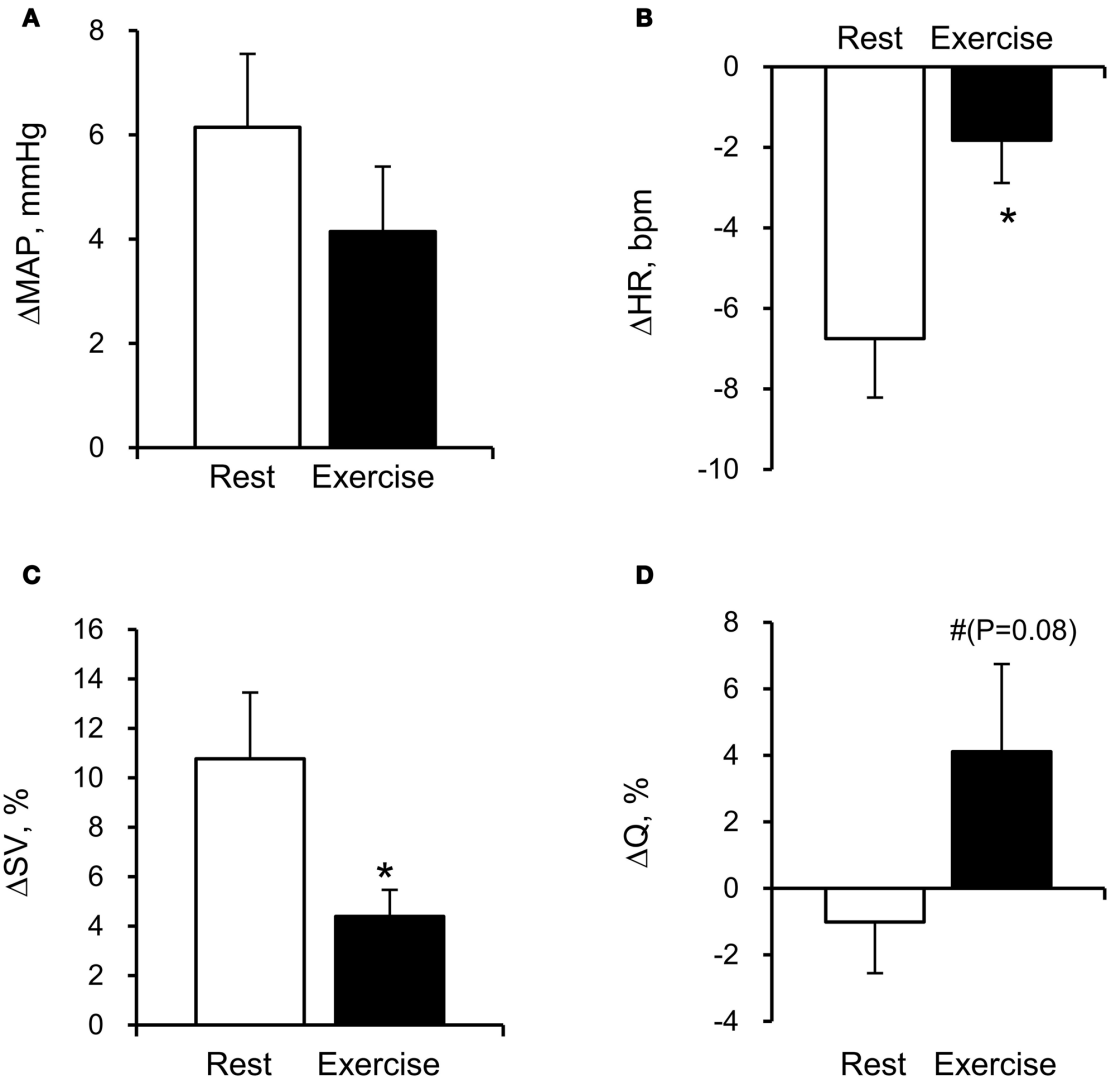
**Facial cooling-induced hemodynamic changes (Δ) at rest and during exercise: (A) mean arterial pressure (MAP), (B) heart rate (HR), (C) stroke volume (SV), and (D) cardiac output (Q).** Values are means ± S.E.M. ^*^P < 0.05, ^#^*P* < 0.1 different from rest.

The average MCA *V*_mean_ was significantly increased by FC (Rest, +1.4 ± 0.5 cm/s; Exercise, +1.4 ± 0.6 cm/s: *P* < 0.05). However, the relation between the FC-evoked changes in MCA *V*_mean_ at rest and during exercise differed among subjects; two subjects showed similar changes, three subjects showed larger responses during exercise; and four subjects showed smaller responses during exercise. The FC-induced decrease in SkBF_face_ was augmented during exercise compared with the resting condition (SkBF_face_: Rest, −32 ± 4%; Exercise, −64 ± 10%, *P* = 0.012; Figures 1 and 2). MAP at rest and during exercise were increased by FC (Rest, +6.2 ± 1.4 mmHg; Exercise, +4.2 ± 1.2 mmHg, *P* < 0.05). However, the changes in MAP in response to FC were not different between at rest or during exercise (*P* = 0.13). The FC-induced decrease in HR and increase in SV were significantly attenuated during exercise compared with the resting condition (HR: Rest, −6.8 ± 1.5 bpm; Exercise, −1.8 ± 1.1 bpm, *P* < 0.05; SV: Rest, +11 ± 3%; Exercise, +4 ± 1%, *P* = 0.015, Figure 3). The FC-induced changes in Q were larger during exercise compared with the resting condition (Rest, −1.0 ± 1.5%; Exercise, +4.1 ± 2.6%, *P* = 0.08). The percent changes in MCA *V*_mean_ induced by FC were not significantly correlated with the percent changes in SkBF_face_ (*r* = 0.095, *P* = 0.709).

## Discussion

The findings of the present study provide new information regarding CBF regulation to FC with water mist (FC) during dynamic exercise. As expected, FC was associated with an increase in MCA *V*_mean_ at rest and during exercise. FC elicited a greater restriction of SkBF_face_ during exercise compared with the resting condition. However, this larger decrease in SkBF_face_ did not result in a larger increase in MCA *V*_mean_ during exercise. In addition, changes in MCA *V*_mean_ with the FC did not correlate with the concomitant changes in SkBF_face_. These findings suggest that other physiological mechanisms apart from decreased SkBF_face_, such as cardiovascular components of the diving reflex, may affect CBF during FC at least during mild dynamic exercise.

Anatomically, the ECA supplies blood to the face, anterior neck, and cranial surface. The ECA blood flow gradually increases with exercise intensity (Sato et al., 2011). In addition, this increase in ECA blood flow significantly correlates with the concomitant change in forehead cutaneous vascular conductance (Sato et al., 2011), suggesting that ECA blood flow is likely increased selectively for thermoregulatory purposes. Similarly, in the present study, SkBF_face_ was increased even during mild exercise as skin temperature rose. Moreover, selective FC decreased SkBF_face_ both at rest and during exercise (Table 2, Figures 1 and 2). This SkBF_face_ response may be due to a similar mechanism seen in the peripheral circulation, e.g., the local cooling-induced vasoconstriction (Johnson and Kellogg, 2010). As expected, the decrease in SkBF_face_ was significantly greater during exercise than that at rest (*P* = 0.012). These different SkBF_face_ responses may reflect an exercise-induced increase in facial capillary volume (+132%, *P* = 0.009) for thermoregulation rather than different autonomic regulation. In any case, these findings provide evidence that exercise-induced heat stress modifies the blood circulation in the head.

The common carotid artery bifurcates into the ECA and ICA; therefore, it is possible that changes in ECA blood flow could affect ICA flow and thus CBF. Indeed, during exercise changes in ECA blood flow were related to changes in ICA blood flow (Sato et al., 2011). Since fatigue and perception of effort during exercise may be related to CBF (Ide and Secher, 2000; Secher et al., 2008; Ogoh and Ainslie, 2009), we hypothesized that FC-induced effects on exercise time to fatigue (Ansley et al., 2008) and lowering of RPE (Mundel et al., 2007; Ansley et al., 2008) in the heat may have been associated with changes in blood flow to the face. In the present study, however, the FC-induced larger decreases in SkBF_face_ but did not lead to proportional changes in MCA *V*_mean_ during exercise (Figure 2). Furthermore, there were no significant correlations between FC-induced changes in MCA *V*_mean_ and SkBF_face_ at rest and during exercise (*P* = 0.709). These results suggest that the FC-induced increase in CBF could not be explained solely by changes in SkBF_face_ at least during mild exercise.

The interaction between facial blood flow and CBF may be mediated by factors associated with exercise intensity or the diving reflex. Sato et al. (2011) demonstrated that the increase in ECA blood flow from moderate to heavy intensity exercise was negatively correlated with the decrease in ICA blood flow. They suggested that a large increase in ECA blood flow contributes to the decrease in ICA blood flow observed during heavy exercise. In the present study, however, the relatively low exercise intensity and thermal stress may have been insufficient to cause significant diversion of blood from the brain (ICA) to the face (ECA). Indeed, FC did not change RPE during exercise in the present study despite an increase in MCA *V*_mean_ (Table 2). During prolonged exercise at higher intensity or ambient temperature, the decrease in CBF, perhaps due to diversion of blood to the face, may still influence the development of central fatigue (Nybo and Nielsen, 2001; Nybo et al., 2002; Dalsgaard et al., 2004). In these conditions, SkBF_face_ largely increases for thermoregulatory purposes and FC may attenuate facial vasodilatation and help preserve CBF.

Another possibility is that FC elicits the diving reflex in which facial cold receptors stimulate cardiovascular changes, including bradycardia and peripheral vasoconstriction (Fagius and Sundlof, 1986; Foster and Sheel, 2005; Kinoshita et al., 2006) that may divert blood toward the brain. For this to occur there would need to be a larger increase in peripheral relative to cerebral vascular resistance. Indeed, the increase in TPR was larger than CVRi at with FC at rest (+7.4% vs. +4.7%). Interestingly, this difference in the FC-induced change between TPR and CVRi was not evident during exercise (+1.3% vs. +1.7%). While CBF is generally believed to be maintained within a narrow range despite changes in MAP between 60 and 150 mmHg—commonly known as cerebral autoregulation (Lassen, 1959). Lucas et al. (2010) indicated that CBF closely follows pharmacological-induced changes in blood pressure in humans. Therefore, it is possible that the increase in MAP induced by FC might have affected the MCA *V*_mean_ response during exercise. Further studies are needed for confirming the possibility. Also, Q affects CBF at rest and during exercise (Ogoh et al., 2005). In the present study, the changes in Q evoked by FC were negligible and not significant at rest and during exercise (Table 2). Therefore, it would appear that the changes in Q had a small effect on the FC-induced increase in CBF. However, higher Q response to FC (*P* = 0.08) may contribute to increase in MCA *V*_mean_ during exercise. In addition, the diving reflex to FC enhances cardiac parasympathetic nerve activity via activation of facial receptors and the trigeminal nerve pathways (Heistad et al., 1968; Khurana et al., 1980). In the present study, FC decreased HR at rest and during exercise. The parasympathetic system has a potential vasodilator effect on brain vessels by mediators such as vasoactive intestinal peptide, acetylcholine, and nitric oxide (Hamel, 2006). More recently, it has been demonstrated that parasympathetic involvement might have a vasodilatory role in the regulation of the cerebral resistance vessels during mild dynamic exercise in humans (Seifert et al., 2010). Thus, it is likely that the increase in CBF evoked by FC might be associated with parasympathetic activation. However, HR responses to FC were different between rest and exercise. Taken together, the relative distribution of blood flow between the brain and the face appears to be regulated by different mechanisms depending on the level of exercise intensity and thermal stressors.

The present study involves some technical considerations. First, the transcranial Doppler ultrasonography-derived MCA *V*_mean_ is an index of regional CBF, and it is acknowledged that this estimate is justified as a measure of CBF only if the vessel diameter is maintained (Valdueza et al., 1997; Serrador et al., 2000). However, the MCA diameter appears to change little during several conditions such as acute hemodynamic perturbations (Giller et al., 1993) and increases in sympathetic activity (Serrador et al., 2000). Additionally, the changes in MCA *V*_mean_ during submaximal dynamic exercise appears to reflect transient changes in CBF determined by other exercise validated techniques [e.g., internal carotid artery blood flow (Hellstrom et al., 1996) and ^133^Xe clearance technique (Jorgensen et al., 1992a,b)]. Second, we used laser Doppler flowmetry to estimate the change in skin blood flow at the forehead. Although this method was used and validated in previous studies for estimation of flow changes in cutaneous regions, this technique has some acknowledged limitations, most notably the inability to provide quantitative measurements of SkBF_face_ in absolute units (Johnson et al., 1984). Therefore, we conducted the rest and exercise trials in the same day and did not move the laser Doppler probes throughout whole experiment to provide quantitative metrics for our comparisons. Finally, the protocol was not designed to identify the mechanism of FC induced-increases in CBF. It is possible that FC-induced hemodynamic changes (i.e., blood pressure) might modify the effects on SkBF_face_ and CBF regulation. Also, it is possible that the contribution of SkBF_face_ to FC induced-changes in MCA *V*_mean_ was altered by exercise itself. Therefore, to address the actual mechanism of FC induced-increases in CBF, further studies, which manipulate SkBF_face_ without altering systemic circulation, will be needed.

## Summary

FC evokes reflexes that reduce SkBF_face_ at rest and to a greater extent during exercise. However, this larger decrease in SkBF_face_ did not result in a larger increase in MCA *V*_mean_ during exercise. Because changes in SkBF_face_ did not correlate with changes in MCA *V*_mean_, FC induced increases in MCA *V*_mean_ during exercise could not be explained solely by changes in SkBF_face_. These results suggest that regulation of skin blood flow during low-intensity exercise has little impact on overall CBF and those other mechanisms.

### Conflict of interest statement

The authors declare that the research was conducted in the absence of any commercial or financial relationships that could be construed as a potential conflict of interest.
